# Acute total sleep deprivation transiently increases plasma total tau but not plasma P-tau181 in healthy adults

**DOI:** 10.3389/fnagi.2026.1904137

**Published:** 2026-08-17

**Authors:** Beiyu Zhao, Meng Wei, Suhang Shang, Ling Gao, Shan Wei, Liangjun Dang, Jie Liu, Chen Chen, Yanbo Li, Jin Wang, Fan Gao, Qiumin Qu

**Affiliations:** 1 Department of Neurology, The First Affiliated Hospital of Xi’an Jiaotong University, Xi’an, China; 2 Clinical Research Center, The First Affiliated Hospital of Xi’an Jiaotong University, Xi’an, China

**Keywords:** acute sleep deprivation, Alzheimer’s disease, plasma p-tau181, plasma t-tau, sleep recovery

## Abstract

**Background:**

Sleep deprivation is a modifiable risk factor for Alzheimer’s disease (AD). Plasma amyloid-β (Aβ) and phosphorylated tau (P-tau) are closely related to cerebral AD pathology and serve as peripheral biomarkers. Our previous work showed that acute sleep deprivation elevated plasma Aβ_40_ in healthy adults. Here we conducted an exploratory analysis to examine the effects of sleep deprivation and subsequent recovery on plasma total tau (T-tau) and P-tau181 levels.

**Methods:**

Twenty healthy adults underwent 24 h of total sleep deprivation followed by daytime naps and a full night of recovery. Venous blood was drawn at 12 predefined time points. Plasma T-tau and P-tau181 were measured by enzyme-linked immunosorbent assay (ELISA).

**Results:**

Plasma T-tau increased by 35.09% (*P* = 0.018) after 24 h of sleep deprivation, and decreased by 37.78% (*P* = 0.005) after sleep recovery. The rise in T-tau was positively correlated with wakefulness duration (β = 0.629, *P* < 0.001). In contrast, no significant changes in plasma P-tau181 were detected with sleep deprivation or recovery under the present experimental conditions.

**Conclusion:**

Under an experimental protocol of 24-h sleep deprivation followed by recovery sleep, transient elevation of plasma T-tau (but not P-tau181) was observed in healthy young adults in this exploratory study. These findings indicate that conditions involving short-term sleep loss are associated with fluctuations in a nonspecific plasma marker of neuronal activity.

## 1 Introduction

Alzheimer’s disease (AD) is an age-related neurodegenerative disease and the leading cause of dementia. Its global prevalence is expected to more than triple by 2050 (Scheltens et al., 2021). The pathological hallmarks of AD include extracellular deposition of amyloid-β (Aβ) plaques and intracellular hyperphosphorylation of tau leading to neurofibrillary tangles, which ultimately drive neurodegeneration and cognitive decline (Busche and Hyman, 2020). Tau pathology, in particular, is closely correlated with the severity of cognitive impairment (Aslanyan et al., 2026), and recent evidence suggests that Aβ accumulation may facilitate the spread of tau pathology across brain regions (Roemer-Cassiano et al., 2025).

Positron emission tomography (PET) imaging of Aβ and tau, along with measurements of cerebrospinal fluid (CSF) Aβ and tau, are established methods for detecting AD pathology (Dubois et al., 2014; Leuzy et al., 2025). However, these approaches are invasive, costly and not easily scalable. In recent years, blood-based biomarkers have emerged as a practical alternative. Accumulating evidence indicates that plasma Aβ and tau correlate with their cerebral levels and with PET-detected brain pathology. Plasma total tau (T-tau) and Aβ have been associated with cognitive decline and brain atrophy (Shi et al., 2019). In a community-based study of middle-aged volunteers, plasma T-tau levels were positively associated with cerebral Aβ deposition (Ramos-Cejudo et al., 2024). However, it should be noted that T-tau is a nonspecific marker that can rise in various conditions associated with acute neuronal stress or injury, and its elevation does not indicate AD-specific pathology (Lennol et al., 2023). By contrast, plasma phosphorylated tau (P-tau) species–particularly P-tau181–are more closely linked to AD-specific neurofibrillary pathology. Findings from a memory clinic cohort further showed that plasma P-tau181 and other P-tau isoforms correlated significantly with Aβ-PET and CSF P-tau181 (Bluma et al., 2025). Likewise, a community-dwelling cohort revealed that plasma Aβ_42_/Aβ_40_ and P-tau181 were markedly altered in Aβ-PET-positive individuals and linked to poorer brain health (Rudolph et al., 2024). These observations have positioned plasma tau and Aβ as accessible tools for studying AD-related pathology (Stevenson-Hoare et al., 2023).

Given the lack of curative treatments for AD, identifying and controlling modifiable risk factors has become a priority. Over the past decade, sleep deprivation has emerged as an important and potentially modifiable risk factor for AD (Ju et al., 2014). Clinical cohort studies linked sleep disorders to cognitive decline and increased brain Aβ burden (Suh et al., 2018; Winer et al., 2020). A meta-analysis of longitudinal studies further supported that short sleep duration was associated with elevated AD risk (Wang et al., 2024). In animal models, chronic sleep deprivation increased Aβ and P-tau levels in the brain and impaired learning and spatial memory (Zhao et al., 2019; Cheng et al., 2023), and chronic sleep disruption was shown to accelerate tau pathology spreading and to interact with synaptic tau burden in promoting cognitive decline (Zhang et al., 2023; Martin et al., 2024). In humans, studies using indwelling lumbar catheters showed that sleep deprivation and the sleep-wake cycle influenced CSF and interstitial fluid (ISF) levels of both Aβ and tau (Kang et al., 2009; Holth et al., 2019). Our previous study in the same cohort found that acute sleep deprivation elevated plasma Aβ_40_ levels in healthy adults, which returned to baseline after sleep recovery (Wei et al., 2017). The present study extends this work by examining the dynamics of plasma tau–specifically T-tau and P-tau181–using the same archived samples, as the effects of sleep deprivation on plasma tau remain incompletely understood. A study in healthy young men reported elevated plasma T-tau after acute sleep deprivation, but blood was collected at only two time points (evening and morning), which did not allow a dynamic assessment across the sleep-wake cycle (Benedict et al., 2020). Another study found that acute sleep deprivation reduced plasma AD biomarkers, but that study performed blood collections every 2 h during normal sleep, which may have affected normal sleep rhythms (Liu et al., 2023).

Against this background, we conducted an exploratory study in 20 healthy adults undergoing 24 h of total sleep deprivation and subsequent sleep recovery, with blood sampled at 12 predefined time points to track the dynamic changes in plasma T-tau and P-tau181.

## 2 Materials and methods

### 2.1 Subjects

Twenty cognitively normal young volunteers were recruited from the local population. Participants provided information on basic physical status, neurological and mental health, and previous medication use. Inclusion criteria were: self-reported good health, no chronic illnesses, normal liver and kidney function, no long-term medication use, regular sleep habits (approximately 8 h per day, bedtime between 22:00 and 23:00, wake-up between 06:00 and 08:00), Pittsburgh Sleep Quality Index (PSQI) < 8 (Zhang et al., 2024), a Chinese Mini-Mental State (CMMS) score ≥ 28 (Katzman et al., 1988), and three regular meals per day. Exclusion criteria included shift work within the past 6 months, smoking, current medication use, nicotine or alcohol intake.

This study reports a secondary analysis of plasma samples collected from a prior registered clinical trial (ChiCTR-OIC-16007975). The original trial was designed to investigate the effects of acute sleep deprivation on plasma Aβ dynamics. All participants provided written informed consent for the collection and storage of plasma for future analyses related to AD pathology. The current measurement of T-tau and P-tau181 represents an exploratory biomarker analysis on these stored samples. The study was conducted in accordance with the Declaration of Helsinki and approved by the Ethics Committee of the First Affiliated Hospital of Xi’an Jiaotong University (No: XJTU1AF2026LSYY-0395).

### 2.2 Study design and procedure

Before the formal experiment, all participants spent one acclimatization day in the laboratory to familiarize themselves with the environment and procedures. During this night, they slept under the same controlled conditions as in the subsequent phases, but no measurements were taken.

The room environment was controlled to mimic the participants’ normal living conditions: ambient temperature was maintained at 22 °C–24 °C, relative humidity at 40%–60%, and lighting followed a simulated day-night cycle (standard indoor illumination from 08:00 to 22:00, dim light thereafter). Standardized meals were provided at 08:30, 12:30, and 17:00, with a snack at 22:00. Each main meal provided approximately 550–900 kcal, with 50%–60% from carbohydrates, 15%–20% from protein, and 20%–30% from fat, conforming to the Chinese Dietary Guidelines. The snack consisted of a piece of fruit or low-calorie yogurt (approximately 100 kcal).

On the morning of the experiment, demographic data and physiological information were recorded from all participants. They then underwent 24 h of total sleep deprivation from 08:00 on day 1 to 08:00 on day 2. During this period, participants remained in the well-lit, temperature-controlled laboratory room under continuous staff supervision. Several measures were taken to ensure wakefulness: participants were engaged in non-strenuous activities (e.g., board games, conversation, watching movies), and regular checks were performed every 30 min with simple questions to confirm alertness. Any attempt to sleep (e.g., reclining in a chair or closing eyes for more than 1 min) was immediately interrupted.

Pre-specified stopping criteria were established before the study: withdrawal at participant request, severe drowsiness unresponsive to intervention, significant psychological distress, any clinically significant adverse event, or any medical condition that, in the investigator’s judgment, would make continued participation unsafe. None of these criteria were triggered. No serious adverse events occurred; participants reported only transient, mild symptoms typical of sleep deprivation (fatigue, sleepiness, mild headache, difficulty concentrating), all of which resolved after recovery sleep.

After the 24-h sleep deprivation, recovery sleep was allowed without restriction: short daytime naps from 09:00 to 11:00 and 13:00 to 15:00 on day 2, followed by a full night of recovery sleep from 22:00 on day 2 to 08:00 on day 3. Participants were not permitted to drive themselves home after the experiment; they arranged for a companion or were provided transport by the research team.

### 2.3 Blood sample collection

**FIGURE 1:**
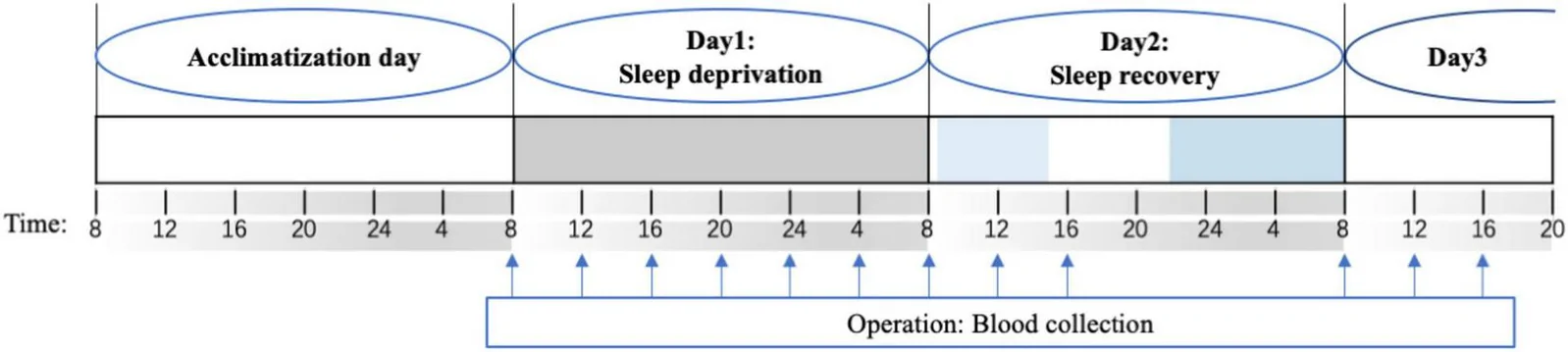
Study timeline and blood collection schedule. After an acclimatization day, participants were sleep-deprived for 24 h (gray vertical bar: 08:00 on day 1 to 08:00 on day 2). Sleep recovery included daytime naps (light blue bar: 09:00–11:00 and 13:00–15:00 on day 2) and a full night (dark blue bar: 22:00 on day 2 to 08:00 on day 3). Blood was collected at 12 time points (indicated by arrows).

Venous blood (3 mL) was collected every 4 h using ethylene diamine tetraacetic acid (EDTA) tubes. To avoid disturbing sleep recovery, no blood draws were performed between 16:00 on day 2 and 08:00 on day 3. Each participant contributed 12 blood samples, of which the 08:00 samples were fasting. Within 2 h of collection, samples were centrifuged at 2000 × *g* for 15 min. Plasma aliquots (200 μL each) were prepared in cryotubes and stored at −80° from collection in 2017 until tau analysis in 2026, without undergoing any freeze-thaw cycles. All samples were visually inspected and none showed hemolysis. The experimental procedure and blood collection time points are shown in Figure 1.

### 2.4 Assessment of plasma T-tau and P-tau181

After thawing to room temperature, plasma concentrations of T-tau and P-tau181 were measured using enzyme-linked immunosorbent assay (ELISA) kits according to the manufacturers’ instructions (Wei et al., 2022). T-tau was measured using a commercially available kit (Cloud-Clone Corp., Wuhan, China; Cat# SEB983Hu, Lot# L250212238). This kit employs a double-antibody sandwich ELISA format targeting microtubule-associated protein tau (MAPT), with a calibration range of 31.2–2,000 pg/mL, a sensitivity of <12.8 pg/mL, intra-assay CV < 10%, and inter-assay CV < 12%, as reported by the manufacturer. P-tau181 was measured using a commercially available kit (Fankewei, Shanghai, China; Cat# F10829-B, Lot# B250714), which targets P-tau181 peptide IPAKTPPAPK(pT)PPSSGEPPK, with a calibration range of 2–64 pg/mL, sensitivity < 0.98 pg/mL, intra-assay CV of 5.02%–6.92%, and inter-assay CV of 7.69%–11.03%. To minimize inter-plate variation, all 12 samples from each participant were assayed on the same ELISA plate. The assays were performed with the laboratory staff blinded to the sampling time point and participant identity. Each sample was measured in duplicate, and quality control samples (three concentrations: low, medium, high) were included on each plate. The intra-assay coefficient of variation (CV) calculated from these quality-controls was ≤10%. Absorbance was read at 450 nm on a Raytor RT-6000 analyzer (Raytor Inc., Shenzhen, China), and concentrations were determined from standard curves.

### 2.5 Statistical analyses

In this study, we examined the fluctuation of plasma tau levels after sleep deprivation and after sleep recovery. Categorical variables are presented as frequencies and percentages. Continuous variables were tested for normality using the Shapiro-Wilk test. Normally distributed data are reported as mean ± standard deviation (SD); non-normally distributed data are presented as medians with interquartile ranges.

To examine changes in plasma T-tau and P-tau181 across the 12 time points, linear mixed-effects models (LMM) were used. Time point (12 levels, categorical) was entered as a fixed effect, and participant-specific random intercepts were included to account for within-subject correlation across repeated measurements; a variance components covariance structure was adopted for the random intercept. Pairwise comparisons between time points were adjusted using the Bonferroni method. Mean differences with 95% confidence intervals are reported for all key pairwise contrasts. For standardized effect sizes, Cohen’s d was calculated for pairwise comparisons. *Post hoc* residual diagnostics were performed to confirm model validity: normality of residuals was assessed using the Shapiro–Wilk test.

To assess the association between plasma tau levels and wakefulness duration during the 24-h sleep deprivation phase (7 time points per participant), another LMM was constructed. Wakefulness duration (hours, continuous) was entered as the fixed effect, with the same random intercept structure. No additional covariates were included, given the small sample size and the homogeneous nature of the cohort.

To assess the robustness of the primary LMM results, we repeated the analysis of time effects on plasma T-tau and P-tau181 using repeated-measures analysis of variance (RM-ANOVA). Sphericity was assessed with Mauchly’s test; when violated, the Greenhouse-Geisser correction was applied. Pairwise comparisons between time points were adjusted with Bonferroni correction. Effect sizes for the RM-ANOVA are reported as partial η^2^.

Statistical analyses were performed using SPSS version 22 (IBM). Graphs were generated with GraphPad Prism 7.0. A two-tailed *P* < 0.05 indicated statistical significance.

## 3 Results

### 3.1 Clinical characteristics of study participants

A total of 20 cognitively normal participants (11 males and 9 females) with a mean age of 27.3 ± 3.4 years were enrolled. They were in good physical condition (BMI: 20.9 ± 2.4 kg/m^2^) and had normal sleep habits (PSQI < 8). Cognitive function assessed by the CMMS was within the normal range (median 30, range 28–30). Across all 12 time points, the overall mean plasma T-tau level was 41.4 ± 13.1 pg/mL and the overall mean plasma P-tau181 level was 33.2 ± 10.1 pg/mL (*n* = 240 measurements from 20 participants). Other demographic and clinical characteristics are summarized in Table 1.

**TABLE 1:** Demographics data of all participants.

Variables	Total (*n* = 20)
Age (year), mean ± SD	27.3 ± 3.4
Male, *n* (%)	11 (55%)
BMI (kg/m^2^), mean ± SD	20.9 ± 2.4
Pulse (bpm), mean ± SD	74.5 ± 10.2
SBP (mmHg), mean ± SD	121.6 ± 6.3
DBP (mmHg), mean ± SD	73.1 ± 9.6
CMMS, median (P25, P75)	30 (29, 30)
PSQI, median (P25, P75)	6.0 (5.0, 7.0)
T-tau (pg/mL), mean ± SD	41.4 ± 13.1
P-tau181 (pg/mL), mean ± SD	33.2 ± 10.1

BMI, body mass index; SBP, systolic blood pressure; DBP, diastolic blood pressure; CMMS, Chinese Mini-Mental State; PSQI, Pittsburgh Sleep Quality Index; T-tau, total tau; P-tau181, phosphorylated tau181.

### 3.2 Changes in plasma tau levels after sleep deprivation

**FIGURE 2:**
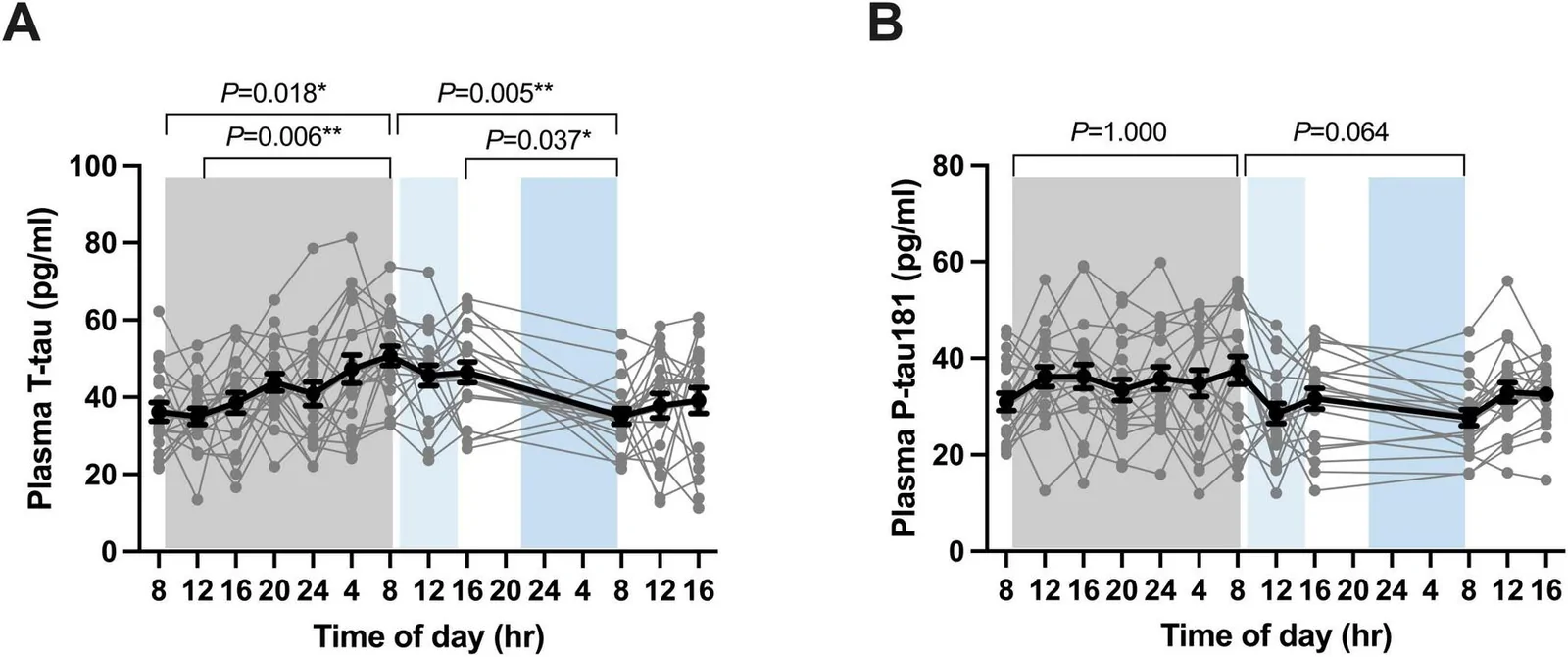
Plasma T-tau and P-tau181 levels over the study period. **(A)** Plasma T-tau. **(B)** Plasma P-tau181. Individual participant trajectories are shown as semi-transparent gray lines (*n* = 20); group mean ± SEM is overlaid as a solid black line. The gray vertical bar indicates the 24-h sleep deprivation period (08:00 on day 1 to 08:00 on day 2). Light and dark blue bars indicate sleep recovery during daytime and night-time, respectively. *P < 0.05, **P < 0.01 for Bonferroni-corrected pairwise comparisons from linear mixed-effects models. T-tau, total tau; P-tau181, phosphorylated tau181.

Figure 2 shows plasma T-tau and P-tau181 levels at the 12 sampling time points, and individual participant trajectories are shown as semi-transparent gray lines, with the group mean ± SEM overlaid as a solid black line. Table 2 summarizes the key comparison of three time points: before sleep deprivation (08:00 on day 1), after sleep deprivation (08:00 on day 2) and after sleep recovery (08:00 on day 3).

**TABLE 2:** Comparison of plasma tau at 08:00 on three separate days.

Comparisons	Mean difference (pg/mL)	95% CI	*P*	Cohen’s d
T-tau				
8:00 on day 2 vs. 8:00 on day 1	14.5	1.1–27.9	0.018*	0.87
8:00 on day 3 vs. 8:00 on day 2	−15.6	−28.8 to −2.5	**0.005^#^**	1.49
P-tau181				
8:00 on day 2 vs. 8:00 on day 1	6.5	−4.1 to 17.2	1.000	0.39
8:00 on day 3 vs. 8:00 on day 2	−9.7	−20.4 to 0.9	0.064	0.45

*After SD vs. before SD, *P* < 0.05; ^#^After SR vs. after SD, *P* < 0.05. SD, sleep deprivation; SR, sleep recovery; T-tau, total-tau; P-tau181, phosphorylated tau181.

For T-tau, the linear mixed model revealed a significant main effect of time point (*F* = 3.083, *P* = 0.001). T-tau levels increased during the first day of sleep deprivation. Following short daytime recovery periods, T-tau showed a numerical decrease at 12:00 and 16:00 on day 2, but the differences were not statistically significant. After the full night of recovery sleep, T-tau decreased significantly. *Post hoc* pairwise comparisons with Bonferroni correction indicated that T-tau peaked at 08:00 after sleep deprivation, higher than baseline (mean difference = 14.5, 95% CI: 1.1–27.9, *P* = 0.018) and higher than the value at 12:00 on day 1 (mean difference = 15.6, 95% CI: 2.3–29.0, *P* = 0.006). After the sleep recovery during night, T-tau reached its lowest level at 08:00 on day 3 (vs. 08:00 on day 2: mean difference = −15.6, 95% CI: −28.8 to −2.5, *P* = 0.005; vs. 16:00 on day 2: mean difference = −11.4, 95% CI: −22.5 to −0.3, *P* = 0.037) (Figure 2A).

For plasma P-tau181, the linear mixed model revealed a significant overall time effect (*F* = 1.951, *P* = 0.034). However, after Bonferroni correction for multiple comparisons, none of the pairwise differences between individual time points reached statistical significance (all adjusted *P* > 0.05). The comparison between after-deprivation and baseline (time point 7 vs. 1) yielded an adjusted *P*-value of 1.00, and the comparison between after-recovery and after-deprivation (time point 10 vs. 7) gave an adjusted *P*-value of 0.064. Thus, while minor fluctuations were present across the sampling points, no robust time-specific changes were identified for plasma P-tau181. We interpret the weak overall temporal fluctuation as most likely reflecting normal physiological diurnal variation rather than a meaningful response to sleep loss (Figure 2B).

### 3.3 Association between plasma tau levels and wakefulness duration

**FIGURE 3:**
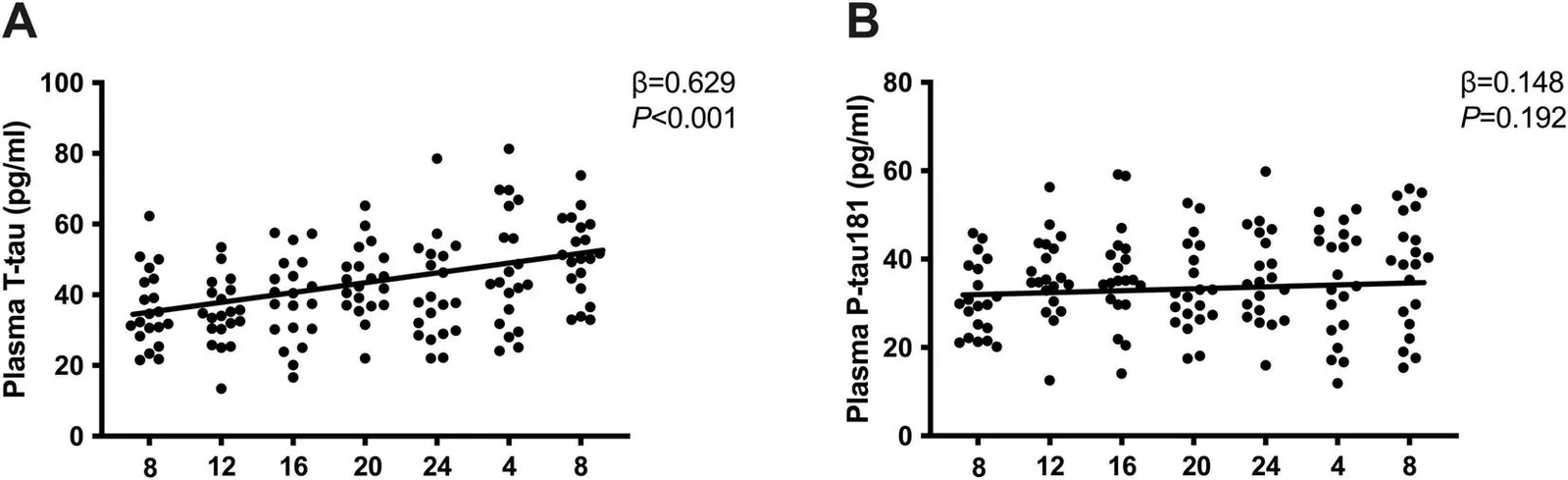
Scatter plots of plasma tau levels against wakefulness duration during the 24-h sleep deprivation phase. **(A)** For plasma T-tau, each additional hour of wakefulness was associated with a significant increase of 0.629 pg/mL (*P* < 0.001). **(B)** For P-tau181, no significant association was observed (β = 0.148, *P* = 0.192). The regression lines are shown for visualization only. Statistical inference was performed using linear mixed-effects models with participant-specific random intercepts. T-tau, total tau; P-tau181, phosphorylated tau-181.

To test the relationship of plasma tau levels and duration of wakefulness, we analyzed data from the 24-h sleep deprivation phase (08:00 on day 1 to 08:00 on day 2) using linear mixed-effects models. For T-tau, the model showed a positive association with wakefulness duration. Each additional hour of wakefulness was associated with an average increase of 0.629 pg/mL in T-tau concentration (β = 0.629, 95% CI: 0.375–0.884, *P* < 0.001). In contrast, plasma P-tau181 was not significantly associated with wakefulness duration (β = 0.148, 95% CI: −0.075 to 0.372, *P* = 0.192). Scatter plots with linear regression fits are shown in Figure 3.

### 3.4 Sensitivity analysis

To assess the robustness of the primary LMM findings, we re-analyzed the data using repeated-measures ANOVA with Greenhouse-Geisser correction (Supplementary Table 1). For plasma T-tau, the overall time effect remained significant [*F* (5.824, 110.648) = 4.385, *P* = 0.001, partial η^2^ = 0.188], representing a large effect size. *Post hoc* pairwise comparisons with Bonferroni correction confirmed that T-tau was significantly higher at post-deprivation (08:00 on day 2) than at 12:00 on day 1 (mean difference = 15.7, 95% CI: 1.8–29.5, *P* = 0.015) and lower after recovery sleep (08:00 on day 3) compared with post-deprivation (mean difference = −15.6, 95% CI: −25.0 to −6.2, *P* < 0.001). The comparison between baseline and post-deprivation did not reach statistical significance after Bonferroni correction (*P* = 0.066). For plasma P-tau181, RM-ANOVA also showed a significant time effect [*F* (5.870, 111.538) = 2.961, *P* = 0.011, partial η^2^ = 0.085]. However, after Bonferroni correction for multiple comparisons, none of the pairwise differences between individual time points reached statistical significance (all adjusted *P* > 0.05) (Supplementary Table 2).

The marginally less significant result for the baseline-versus-post-deprivation comparison in RM-ANOVA (adjusted *P* = 0.066) compared with LMM (adjusted *P* = 0.018) reflects methodological differences between the two frameworks rather than conflicting biological signals. LMM leverages all 12 time points to estimate the within-subject covariance structure, yielding higher statistical power, whereas the Greenhouse-Geisser correction in RM-ANOVA reduces effective degrees of freedom and produces a more conservative test. Importantly, the mean difference between baseline and post-deprivation was 14.5 pg/mL with a Cohen’s d of 0.87 in the primary analysis, indicating a large effect size. Thus, this contrast is best interpreted as a real biological trend that narrowly missed the conventional significance threshold in the more conservative RM-ANOVA. Critically, the recovery effect (post-recovery vs. post-deprivation) was statistically significant in both analyses (LMM: *P* = 0.005; RM-ANOVA: *P* < 0.001), confirming the robustness of the core finding that T-tau rises during sustained wakefulness and falls after recovery sleep. We therefore consider our main conclusions adequately supported, while acknowledging that the baseline to post-deprivation comparison should be interpreted with some caution.

## 4 Discussion

In this exploratory study of 20 healthy young adults undergoing a 24-h total wakefulness protocol followed by structured recovery sleep, we observed a transient increase in plasma T-tau levels during prolonged wakefulness, which returned toward baseline after subsequent sleep recovery. The increase in T-tau correlated with the duration of wakefulness. In contrast, plasma P-tau181 showed no significant changes under the same conditions. These findings indicate that under the experimental conditions of prolonged wakefulness and recovery, plasma T-tau–a nonspecific marker of neuronal activity–exhibited transient fluctuations, whereas the more AD-specific marker P-tau181 remained stable.

Most prior work linking sleep deprivation to tau has focused on CSF and brain ISF. Holth et al. (2019) showed that ISF tau in mice rose by roughly 90% during wakefulness and 100% during sleep deprivation compared with sleep, and that CSF tau in healthy adults increased by more than 50% after sleep deprivation. These findings clearly demonstrate that the sleep-wake cycle and sleep deprivation affect tau levels in the ISF and CSF. However, plasma and CSF tau differ in kinetics and clearance pathways, so direct comparisons between their dynamics require caution. Two recent landmark studies have examined plasma tau responses to sleep deprivation, with results that initially seem at odds with ours. Liu et al. (2023) reported that CSF Aβ, unphosphorylated tau (T181, T217), and phosphorylated tau (pT181) increased by 35%–55% during sleep deprivation, whereas plasma concentrations of the same markers fell by 5%–15%, leading to the interpretation that sleep deprivation reduces CSF-to-blood clearance of AD-related biomarkers. Similarly, Dagum et al. (2026) showed that glymphatic clearance during normal sleep raised morning plasma levels of AD biomarkers compared with sleep deprivation. Against this backdrop, our data indicate that acute sleep deprivation significantly elevates plasma T-tau in a manner that correlates with wake duration, and that this elevation reverses after sleep recovery–at least in the short term. These observations are consistent with a prior report that sleep deprivation increased the evening-morning ratio of plasma T-tau in young men (Benedict et al., 2020). Similarly, patients with obstructive sleep apnea, who experienced chronic sleep fragmentation and poor sleep quality, were reported to have higher plasma T-tau levels than healthy controls (Pai et al., 2022). It is important to note, however, that T-tau is not specific to AD pathology; its elevation can occur in various conditions associated with acute neuronal stress or injury, and should not be equated with AD-specific neurodegenerative changes (Deters et al., 2017; Pase et al., 2019).

The half-life of tau in the brain is relatively long, up to 20 h, but in the peripheral circulation it is only a few minutes (Yanamandra et al., 2017). Therefore, the increase in plasma T-tau during sleep deprivation is unlikely to reflect impaired clearance; a more plausible explanation is that release is enhanced. One mechanism is that prolonged wakefulness keeps neurons in a state of sustained high activity, and increased neuronal activity can rapidly elevate extracellular tau *in vivo* (Yamada et al., 2014; Wang and Holtzman, 2020). Another possibility may be that sleep deprivation triggers neuroinflammation and peripheral inflammatory response, leading to non-specific release of T-tau from both CNS and peripheral tissues (Zetterberg et al., 2013; Stukas et al., 2019; Liu, 2025). Canet et al. (2025) further showed that the rise in body temperature during wakefulness and sleep deprivation was correlated with elevations in both CSF and plasma tau. Collectively, these mechanisms point to enhanced release–from both neuronal sources within the CNS and non-specific peripheral tissues under stress–as the principal driver of increased plasma T-tau during sleep loss. This raises an obvious question: How do these results square with recent evidence that sleep deprivation reduces CNS-to-blood clearance and lowers plasma levels of certain tau species? If sleep deprivation simultaneously boosts neuronal tau release and impairs efflux from the CNS, the net effect on plasma levels would depend on the balance between these opposing processes. Our observation that non-phosphorylated total tau increased suggests that, for this specific species, the combined release from both CNS and peripheral sources may exceed the reduction in clearance. This interpretation is supported by the very short half-life of tau in the peripheral circulation–minutes rather than hours–which means that a sustained elevation must reflect ongoing release rather than passive accumulation. Species specificity likely matters as well: Liu and colleagues measured distinct unphosphorylated and phosphorylated tau species using Simoa, whereas we measured total tau by conventional ELISA. Different tau species may be differentially affected by release versus clearance mechanisms. Methodological differences in assay sensitivity and sampling timing, together with differences in study populations, may also contribute to the discrepancies. The reduction in plasma T-tau after sleep recovery aligns with studies showing that slow-wave sleep facilitates tau clearance from the brain. A randomized crossover study in healthy adults found that CSF levels of T-tau were consistently lower after a night of sleep compared with sleep deprivation or daytime sampling, supporting a model in which slow-wave sleep selectively reduces CSF tau concentrations through enhanced solute mobility and receptor-mediated clearance (Lyckenvik et al., 2025). Given the bidirectional exchange between CSF and plasma, the observed decline in plasma T-tau after recovery sleep may partially reflect this sleep-active clearance mechanism. It should be noted, however, that these mechanistic interpretations remain speculative, as the sleep/wake states were confirmed behaviorally rather than objectively.

In contrast to T-tau, we found no significant change in plasma P-tau181 after 24 h of sleep deprivation, nor any correlation with wakefulness duration. Animal study showed that sleep deprivation increased P-tau in the rat brain (Hitrec et al., 2024). A clinical study reported that sleep deprivation increased CSF T-tau and P-tau181 in healthy adults, with the effects on P-tau being site-specific (Barthelemy et al., 2020). However, studies focusing on plasma yielded different results. It should be noted that CSF and plasma P-tau181 measurements reflect different physiological compartments with distinct kinetics, and discrepancies between CSF and plasma findings do not necessarily indicate contradictory results. A previous study in healthy adults reported that acute sleep deprivation produced a significant decrease in plasma P-tau181 levels (Liu et al., 2023). More recently, Eide et al. (2023) measured plasma P-tau181 after one night of total sleep deprivation and found no significant alteration compared with normal sleep. The absence of measurable plasma P-tau181 response in our study could be explained by several factors. The glymphatic system is a major route for clearing metabolites from the brain, and sleep deprivation impairs glymphatic function, which might reduce efflux of P-tau into the bloodstream (Reddy and van der Werf, 2020; Buccellato et al., 2022). Alternatively, given that plasma P-tau181 exhibits low intra-individual variability and is robust to circadian fluctuations, the absence of measurable change under our experimental conditions may be consistent with its stability as a reliable diagnostic marker for AD pathology (Karikari et al., 2021; Orduna Dolado et al., 2024).

Several limitations should be acknowledged. First, our participants were young and healthy, and the sample size was modest (*n* = 20). While the within-subject repeated-measures design improves statistical power for detecting within-individual changes, the small sample size limits the robustness and generalizability of our findings. Although prospective cohort studies have shown that plasma T-tau levels predict future AD risk (Chen et al., 2019; Pase et al., 2019), larger studies in older adults or individuals with chronic sleep disturbance are needed to confirm our findings. Second, plasma T-tau and P-tau181 were measured using conventional ELISA platforms. While these assays demonstrated acceptable intra-assay precision and dilution linearity in our hands, ELISA is inherently less sensitive than more advanced platforms such as Simoa or automated immunoassays, and its results are more dependent on specific reagent lots and manufacturers. Consequently, absolute concentration values obtained with different ELISA kits–or even different batches of the same kit–may not be directly comparable across laboratories or studies. Notably, this limitation primarily affects cross-study comparisons of absolute values rather than the within-subject temporal dynamics; the 12 repeated measurements per participant were performed on the same plate using the same reagents, so the observed fluctuation patterns–particularly the rise in T-tau during wakefulness and its decline after recovery–remain internally valid and interpretable. Meanwhile, future studies using more extensively validated platforms are required. Third, sleep status was monitored behaviorally through direct supervision and regular alertness checks, rather than by objective methods such as polysomnography (PSG), electroencephalography (EEG), or actigraphy. Therefore, we cannot exclude the possibility of microsleeps, drowsiness, or N1 sleep intrusions during the deprivation period, nor can we determine whether the decline in T-tau after recovery sleep reflects recovery sleep itself, the passage of time, or both, as we cannot characterize the architecture of recovery sleep. Consequently, our interpretations are framed with appropriate caution, and we have avoided mechanistic claims that imply objectively confirmed sleep deprivation or recovery sleep effects. Future studies incorporating PSG/EEG monitoring or actigraphy, will be essential to confirm and extend our findings. Fourth, this study used a within-subject design without a parallel habitual-sleep control arm or crossover normal-sleep condition, and no objective circadian phase marker (e.g., melatonin or core body temperature) was measured. Previous studies have demonstrated that tau levels in CSF and ISF are regulated by the sleep-wake cycle and exhibit circadian fluctuations, and that body temperature changes across the sleep-wake cycle correlate with both CSF and plasma tau elevations (Holth et al., 2019; Canet et al., 2025). In addition, hydration status and physiological stress may all change during prolonged wakefulness (Kamperis et al., 2010; Malik et al., 2025) and could potentially influence plasma biomarker concentrations; these variables were not measured in the present study. Therefore, we cannot fully disentangle the effects of sleep deprivation *per se* from circadian timing, body temperature rhythms, repeated sampling, or other physiological covariates. Accordingly, the observed plasma T-tau changes should be interpreted as associated with the combined experimental condition of prolonged wakefulness and recovery sleep, rather than a definitive causal effect of acute sleep loss alone.

In summary, under our experimental protocol combining 24-h prolonged wakefulness and recovery sleep, plasma T-tau (but not P-tau181) exhibited transient, wakefulness-associated changes in healthy young adults. These findings are exploratory and indicate that conditions involving short-term sleep loss are associated with fluctuations in a nonspecific plasma marker of neuronal activity, but do not establish a definitive causal link between sleep loss and AD pathology. Nonetheless, recent sleep history should be accounted for when interpreting plasma T-tau levels in clinical and research settings.

## Data Availability

The raw data supporting the conclusions of this article will be made available by the authors, without undue reservation.
