# Supplementary material for: Acute total sleep deprivation transiently increases plasma total tau but not plasma P-tau181 in healthy adults
Source: Front Aging Neurosci. 2026 Aug 17;18:1904137. doi: 10.3389/fnagi.2026.1904137 (PMC13527036; doi:10.3389/fnagi.2026.1904137)
Supplement: Supplementary file 1 [file Table_1.docx]

**Supplementary Table S1. Sensitivity analysis for plasma T‑tau (RM‑ANOVA with Bonferroni correction).**

| **Comparisons** | **Mean difference (pg/mL)** | **95% CI** | ***P*** |
| --- | --- | --- | --- |
| 8:00 on day 2 vs. 8:00 on day 1 | 14.523 | -0.442 – 29.488 | 0.066 |
| 8:00 on day 2 vs. 12:00 on day 1 | 15.672 | 1.830 – 29.515 | **0.015** |
| 8:00 on day 3 vs. 8:00 on day 2 | -15.635 | -25.029 – -6.242 | **<0.001** |
| 8:00 on day 3 vs. 16:00 on day 2 | -11.401 | -23.996 – 1.195 | 0.119 |

Key comparisons from the primary analysis are shown.

**Supplementary Table S2. Sensitivity analysis for plasma P‑tau181 (RM‑ANOVA with Bonferroni correction).**

| **Comparisons** | **Mean difference (pg/mL)** | **95% CI** | ***P*** |
| --- | --- | --- | --- |
| 8:00 on day 2 vs. 8:00 on day 1 | 6.530 | -21.489 – 8.429 | 1.000 |
| 8:00 on day 3 vs. 8:00 on day 2 | -9.758 | -22.145 – 2.630 | 0.344 |

None of the pairwise comparisons reached statistical significance after Bonferroni correction.
